# Differential effects of the recombinant type 1 ribosome-inactivating protein, OsRIP1, on growth of PSB-D and BY-2 cells

**DOI:** 10.3389/fpls.2022.1019591

**Published:** 2022-09-29

**Authors:** Simin Chen, Koen Gistelinck, Isabel Verbeke, Els J. M. Van Damme

**Affiliations:** ^1^ Department of Biotechnology, Faculty of Bioscience Engineering, Ghent University, Ghent, Belgium; ^2^ Center for Advanced Light Microscopy, Ghent University, Ghent, Belgium

**Keywords:** ribosome-inactivating protein, BY-2 cells, PSB-D cells, cell death, exogenous application

## Abstract

Plant suspension cells were treated with recombinant OsRIP1, a type 1 ribosome-inactivating protein (RIP) from rice (*Oryza sativa* L.). OsRIP1 triggered cell death in tobacco BY-2 cells but not in *Arabidopsis* PSB-D cells. Phenotypic changes in BY-2 cells exposed to OsRIP1, included loss of growth capacity, loss of integrity of the plasma membrane and vacuolar collapse. These effects were also accompanied by RNA degradation and DNA fragmentation. Targeting of exogenous OsRIP1 to plant vacuoles and OsRIP1-induced accumulation of transcripts for vacuolar processing enzymes (VPEs) indicated that OsRIP1 provoked plant cell death in tobacco BY-2 cells through the activation of VPEs and subsequent vacuolar disruption, which was probably independent of its *N*-glycosylase activity on cytosolic ribosomes. Necrosis with limited production of H_2_O_2_ was observed after infiltration of high concentrations of OsRIP1 in epidermal cells of *Nicotiana tabacum* cv. Samsun NN plants. Our study provides the first evidence that OsRIP1 exerts differential effects on the growth of PSB-D and BY-2 cells. The vacuole-dependent cell death pathway is associated with the lethal effect of the exogenously applied OsRIP1 on BY-2 cells.

## 1 Introduction

Plants contain a huge amount of physiologically active phytochemicals affecting various intracellular processes (Surguchov et al., 2021). Ribosome-inactivating proteins (RIPs) are a class of cytotoxic enzymes [EC 3.2.2.22] capable of depurinating the 28S ribosomal RNA because of their *N*-glycosylase activity, thus blocking translation in an irreversible manner. RIPs, being a group of plant toxins, are prevalent in many plant species although in highly variable concentrations (Wong et al., 2020). RIPs function as RNA *N*-glycosylases and can be classified into two major groups based on their molecular structures. Type 1 RIPs are single chain proteins consisting of an active domain of approximately 30 kDa with *N*-glycosylase activity. In contrast, type 2 RIPs comprise two chains, the chain with RIP activity is conjugated to a lectin chain through a disulfide bond (Bolognesi et al., 2016). RIPs synthesized with a leader signal peptide are generally targeted to vacuoles or to the apoplast, and hence are sequestered from the ribosomes of host cells, as demonstrated in great detail for the type 1 RIPs saporin and pokeweed antiviral proteins, as well as the type 2 RIP ricin (Marshall et al., 2011). Other RIPs lacking a signal peptide reside in the cytosol where they could interact with cytosolic ribosomes. Although most of RIPs are inactive to host ribosomes under normal conditions, certain stresses can also induce their activity to depurinate plant ribosomes, as exemplified by tritin-S from wheat (Sawasaki et al., 2008) and JIP60 from barley (Rustgi et al., 2014).

Research has focused a lot on the role of RIPs in plant defense against viruses and fungi. The anti-viral properties of RIPs are attributed to the fact that viral infection facilitates RIPs to traffic from the apoplastic space to the cytosol of plant cells, in which RIPs get access to the ribosomes, exert their enzymatic activity, arrest the protein synthesis and eventually inhibit replication of plant viruses (Citores et al., 2021). Furthermore, RIPs have been recognized to act directly on viral nucleic acids, abolishing their infectivity (Iglesias et al., 2016).

Cereal RIPs are a distinct class of RIPs, as they retain characteristic features and have diversified significantly during evolution. A total of 38 RIP sequences have been identified in the genome sequence of *Oryza sativa* spp. *japonica*, the majority of which does not possess a signal peptide (Wytynck et al., 2017). These rice RIPs, as shown for the RIP genes of LOC_Os01g06740 (OsRIP1) (De Zaeytijd, 2019) and LOC_Os01g07300 (Wytynck, 2020), locate to the same compartment as the ribosomes. This raises some questions about the physiological importance of cytoplasmic cereal RIPs. Some cereal RIPs show little activity against plant ribosomes, while other cereal RIPs exert their functions by interacting with the ribosomes of the host plant. For example, a cytosolic protein, RIP30 from barley is probably only weakly active or completely inactive on host ribosomes (Leah et al., 1991). A cytosolic albumin, b-32 from maize is present in the endosperm as an inactive zymogen (proRIP), and plays an important role in the accumulation of zeins (Barbieri et al., 1997). Wheat RIP, tritin-S, is a cytosolic type 1 RIP from wheat germ and was inactive on host ribosomes. Tritin-S is produced in senescent coleoptiles and plays an important role in the senescing of these coleoptiles (Sawasaki et al., 2008). Similarly, JIP60 (a 60 kDa jasmonate-induced protein) from barley functions as a molecular switch to alter the translational machinery under stress conditions (Rustgi et al., 2014). Overexpression of OsRIP1 in rice yielded transgenic plants did not show any abnormal phenotype, implying that OsRIP1 is not toxic to rice cells (Wytynck et al., 2021).

Battelli et al. (1984) reported the differential effects of RIPs on plant cell growth and showed that ricin stimulated the growth of carrot cells in culture, whereas the pokeweed anti-viral protein from seeds (PAP-S) inhibited the cell growth. However, only few studies report on the cytotoxicity of RIPs for plant cell lines. We investigated the effects of OsRIP1 on tobacco BY-2 cells and *Arabidopsis* PSB-D cell cultures. This study helps to unravel whether a cytosolic RIP affects the growth of heterologous plant cell cultures. Our study shows for the first time a comparative study of *Arabidopsis* PSB-D cells and tobacco BY-2 cells in response to exogenously applied recombinant OsRIP1 produced by *Escherichia coli* (*E. coli*). These two plant cell lines were selected based on their remarkable growth rate and high homogeneity (Develtere, 2019; Mahjouri et al., 2020). OsRIP1-induced phenotypic changes on cell cultures were analyzed, including cell viability, cell density and the integrity of the plasma membrane. Furthermore, OsRIP1 uptake and localization were assessed in tobacco and *Arabidopsis* cell cultures. The integrity of both RNA and DNA were examined in OsRIP1-treated BY-2 cells. Relative mRNA levels for genes encoding vacuolar processing enzymes (VPEs) and genes related to defense and oxidative stress were also quantified.

## 2 Materials and methods

### 2.1 Plant materials

*Nicotiana tabacum* cv. Bright yellow 2 (BY-2) (Nagata et al., 1992) cell suspensions were cultured in liquid MS medium [4.3 g/l Murashige and Skoog salts (MP BIOMEDICALS, Solon, OH), 0.2 g/l KH_2_PO_4_, 30 g/l sucrose, 0.4 mg/l 2,4-D, 1 mg/l thiamine, and 100 mg/l myo-inositol, pH 5.7 (KOH)]. *Arabidopsis* PSB-D cell suspensions were grown in MSMO medium [4.43 g/l MSMO (minimal organics Sigma #M6899), 30 g/l sucrose, 0.5 mg/l NAA and 0.05 mg/l kinetin, pH 5.7 (KOH)]. All cell cultures were grown at 25°C in darkness on a rotary shaker at 130 rpm. BY-2 and PSB-D cell suspensions were sub-cultured weekly at 1:40 and 1:10 dilution ratio, respectively.

Tobacco plants (*Nicotiana tabacum* cv. Samsun NN) were used in the study and grown under a 16 h light/8 h dark photoperiod at constant 28°C with 70% relative humidity.

### 2.2 Recombinant production and purification of OsRIP1

Recombinant OsRIP1 was produced in *E. coli* strain Rosetta (DE3) at 14°C for 72 h and purified as described previously (De Zaeytijd et al., 2019) with minor adaptation. The purified recombinant OsRIP1 was dialyzed against 50 mM potassium phosphate buffer [3.79 g/l K_2_HPO_4_, 3.84 g/l KH_2_PO_4_, pH 6.7 (KOH)].

### 2.3 Treatment of plant cell cultures with OsRIP1

To assess OsRIP1 cytotoxicity, plant cells at the phase of exponential growth (day 3 after subculturing) were subjected to different concentrations of OsRIP1 (0.219-7 μM) at 1:1 volume ratio. Subsequently cells were cultivated in 24-well plates for 96 h, with samples being harvested at strictly defined time intervals of 24, 48, 72 and 96 h. For the control treatment, cells were exposed to 50 mM potassium phosphate buffer (pH 6.7). Samples were snap-frozen in liquid nitrogen before storage at -80°C for later assays. All experiments were carried out at least in triplicates.

### 2.4 OsRIP1 infiltration in tobacco plants

The leaves of 7-week-old tobacco plants were infiltrated with different samples using a syringe without a needle to cover areas of approximately 1 cm^2^. A range of OsRIP1 concentrations (1 μM, 2.5 μM, 5 μM, 10 μM, 30 μM) were examined in order to determine the minimum concentration of OsRIP1 that caused a hypersensitive (HR) response. In the control treatment, the leaves were infiltrated with 30 μM bovine serum albumin (BSA) or potassium phosphate buffer. The necrotic lesions were examined at 24 h, 48 h, 72 h and 96 h post infiltration (Keerio et al., 2020), respectively.

### 2.5 Phenotypic analysis

#### 2.5.1 Cell viability and cell density

The viability of plant cells was determined using PrestoBlue™ Cell Viability Reagent (Invitrogen; Thermo Fisher Scientific, Inc.) according to the instructions. In brief, 10 μl PrestoBlue™ reagent was pipetted to 90 μl of cells in 96-well flat-bottomed plates and incubated for 1 h at 25°C in the dark prior to fluorescence measurement at Ex/Em of 560/590 nm in a plate reader (Infinite 200, Tecan, Mannedorf, Switzerland). The results showed cell viability after treatment with OsRIP1 relative to control cells treated with 50 mM potassium phosphate buffer. Cell density was determined by measuring the absorbance at 600 nm using a Bio-Rad SmartSpec™3000 Spectrophotometer (Bio-Rad, CA, USA) at indicated time intervals.

#### 2.5.2 Loss of the integrity of cell membrane and vacuole staining

The integrity of the plasma membrane was assessed (Poborilova et al., 2013). Briefly, BY-2 cells were washed three times with the MS medium and resuspended in 2 ml of MS medium containing 0.05% (w/v) Evans blue and incubated at 25°C for 15 min. Subsequently, cells were precipitated by centrifugation (150 g, 3 min) and washed with MS medium. Washing steps were repeated until the supernatant after centrifugation was clear. In addition, neutral red staining for intact vacuole was performed following a previously described method (Kariya et al., 2013). Control or OsRIP1-treated cells were incubated with 0.015% neutral red solution for 30 min in the dark, and the vacuole was stained by neutral red and examined under the Widefield microscope Nikon eclipse Ti (Nikon instruments, Badhoevedorp, Netherlands) using a 10x dry objective lens.

### 2.6 Confocal microscopy

OsRIP1 labelling with fluorescein isothiocyanate (FITC) (Sigma-Aldrich) was carried out as described previously (Chen et al., 2021). After incubation with OsRIP1-FITC at desired timepoints, PSB-D cells or BY-2 cells were washed with corresponding medium and observed under a fluorescence microscope (Nikon, Melville, NY, USA) with a filter set Ex488/Em525.

### 2.7 ROS production in tobacco leaves and cell cultures

The histological localization of the H_2_O_2_ in tobacco leaves was detected as reported by Chen et al. (2012). In brief, 7-week-old tobacco leaves were infiltrated with different concentrations of OsRIP1, or with 50 mM potassium phosphate buffer as a control for 24 h. Detached tobacco leaves were incubated with 3,3’-diaminobenzidine (DAB)-HCl (1 mg/ml, pH 3.8) solution at 28°C overnight. Subsequently, the chlorophyll was removed from the leaves by soaking in boiling ethanol (95%) for 20 min, and H_2_O_2_ was visualized as a reddish-brown coloration (Keerio et al., 2020).

Determination of H_2_O_2_ production in cell cultures was conducted according to the previous method (Velikova et al., 2000). Briefly, cell suspensions (500 mg) were homogenized with 5 ml 0.1% (w/v) trichloroacetic acid on ice. After centrifugation at 12,000 g for 15 min at 4°C, 0.5 ml of the supernatant was added to 0.5 ml of 10 mM potassium phosphate buffer (pH 7.0) and 1 ml of 1 M KI, prior to recording the absorbance of the supernatant at 390 nm. The H_2_O_2_ content was expressed in μmol H_2_O_2_ per gram fresh weight (μmol g^-1^ FW) of suspended cells after calibration using a standard curve measured by adding H_2_O_2_ to cell suspension aliquots. BY-2 cells treated with 100 mM NaCl were used as a positive control for the determination of intracellular H_2_O_2_ levels (Jia et al., 2020).

### 2.8 Evaluation of genomic DNA integrity

Total genomic DNA was isolated from control or treated cells following the cetyl trimethylammonium bromide (CTAB)-based protocol (Saghai-Maroof et al., 1984). After RNase A (Thermo Scientific) treatment, DNA concentration and quality were determined by observing the absorbance at 260 nm and 280 nm, and calculating the A260/A280, A260/A230 ratios using the NanoDrop 2000 spectrophotometer (Thermo Scientific). DNA electrophoresis was carried out on a 1.5% agarose gel, followed by visualization in the presence of ethidium bromide. The gel was scanned with a gel imager (Bio-Rad). For quantitative analysis, the band densities of individual regions in the gel were calculated using Fiji (ImageJ, version 1.53c) in grayscale mode to obtain integrated grey values.

### 2.9 RNA extraction and gene expression analysis

Total RNA was extracted from control (CTRL) BY-2 cells or cells treated with OsRIP1, using the Spectrum™ Plant Total RNA Kit (Sigma Life Science). After DNAse treatment, the quantity and quality of the extracted RNA samples were assessed by the NanoDrop 2000 spectrophotometer (Thermo Scientific). To verify the RNA integrity, 1 μg total RNA samples were assessed by horizontal electrophoresis using a 1.0% agarose gel.

Transcript levels for selected genes were determined by reverse transcription quantitative polymerase chain reaction (RT-qPCR) as described previously (Chen et al., 2021). Three biological and two technical replicates were performed for each analysis. Actin-7 and elongation factor 1-α (EF1α) served as internal controls. The expression of each gene of interest was analyzed and normalized to that of the reference genes by qBase+ software (Biogazelle, Ghent, Belgium). Primers used are listed in **Table S1**.

### 2.10 Statistical analysis

Quantitative data are presented as mean± SD of experiments repeated at least three times. One way ANOVA and Duncan test were performed to analyze the data of cell viability and necrotic area (%). Multiple *t*-test was used to evaluate differences in the expression of the genes of interest in BY-2 cells determined by RT-qPCR.

## 3 Results

### 3.1 OsRIP1 reduced the growth of BY-2 cells but not of PSB-D cells

**Figure 1:**
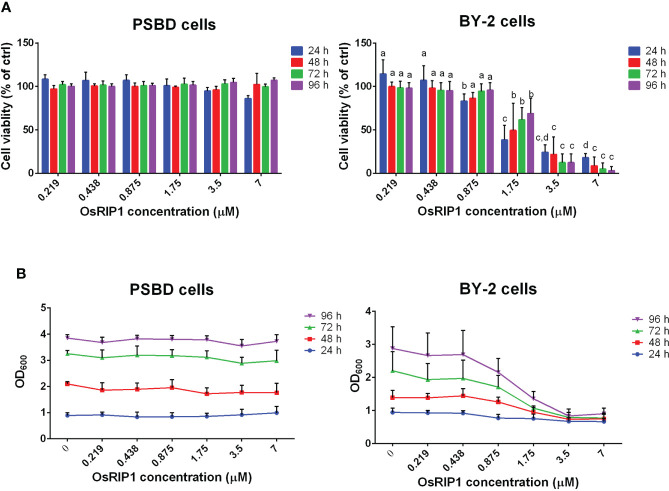
OsRIP1 dose effects on PSB-D cells and BY-2 cells. Cells were incubated in the absence or presence of various concentrations of OsRIP1 for 24 h, 48 h, 72 h and 96 h, respectively. **(A)** cell viability relative to control cells. **(B)** Optical density of cell cultures at 600 nm (OD_600_). 1, PSB-D cells; 2, BY-2 cells. Values are the means ± SD of four independent experiments. Statistically significant differences (p < 0.05) between different concentrations of OsRIP1 treatments after incubation for the same duration (i.e., 24 h, 48 h, 72 h, or 96 h) in the graph are indicated with different letters; the same letters indicate no significant differences, with a>b>c>d.

Cell viability was assessed after exposing PSB-D cells and BY-2 cells to different concentrations of OsRIP1 for 24-96 h. Cell viability of PSB-D cells decreased to 86% after treatment with 7 μM OsRIP1 for 24 h, but then increased to 107% at 96 h post treatment (**Figure 1 A**). No significant differences in cell viability of PSB-D cells were observed after treatment with 0-7 μM OsRIP1 over time. Cell density of PSB-D cells plateaued at 24 h with the value of OD_600_ close to 1, while the OD_600_ of the cell culture was close to 4 at 96 h independent of the OsRIP1 concentrations applied to the cells (**Figure 1B**).

Conversely, when BY-2 cells were subjected to increasing doses of OsRIP1, cell viability and the OD_600_ value of the cell culture decreased, suggesting OsRIP1 toxicity to BY-2 cells in a dose-dependent manner. The cell viability decreased from 83% (0.875 μM OsRIP1) to 18% (7 μM OsRIP1) after incubation for 24 h, and was almost completely lost (3.11%) at 7 μM OsRIP1 at 96 h post treatment (**Figure 1A**). Higher concentrations of OsRIP1 (3.5-7 μM) resulted in a strong decrease in the viability of BY-2 cells at 24 h, and even lower cell viability after 48-96 h incubation time (**Figure 1A**).

No statistically significant differences were found between the viability of BY-2 cells treated with 3.5 μM OsRIP1 and 7 μM OsRIP1 for 24 h, 48 h, 72 h, or even 96 h, nor for the viability of BY-2 cells treated with low concentrations of OsRIP1 (0.219-0.875 μM) for 48-96 h (**Figure 1** panel A2). Accordingly, cell density of BY-2 cells exposed to 0-0.438 μM OsRIP1 increased with incubation time (24-96 h), whereas a less pronounced increase in cell density with time for cells treated with 0.875-1.75 μM OsRIP1 was found. Moreover, almost no increase in cell density was observed when BY-2 cells were subjected to 3.5-7 μM OsRIP1 for 24-96 h (**Figure 1B**).

### 3.2 OsRIP1 was internalized in plant cells

**Figure 2:**
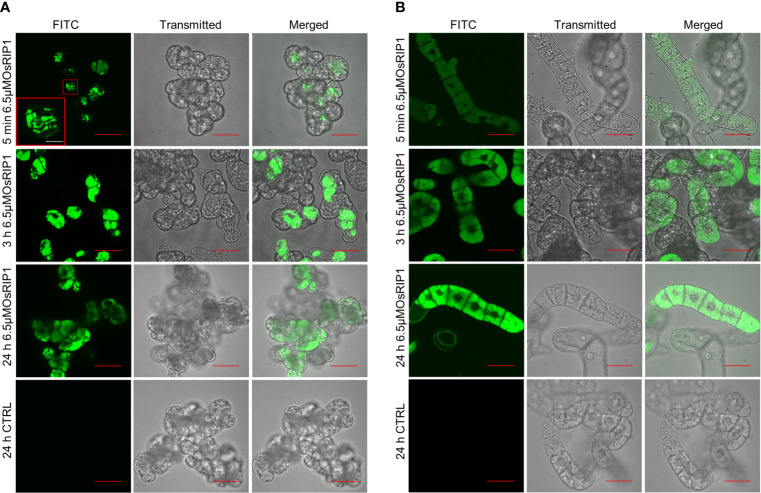
Confocal imaging of OsRIP1 accumulation in PSB-D cells **(A)** and BY-2 cells **(B)** after 5 min, 3 h and 24 h treatment with 6.5 μM OsRIP1 conjugated with FITC using a filter set Ex488/Em532.5. All scale bars in red indicate 50 μm, while the white bar refers to 10 μm.

The uptake and localization of OsRIP1-FITC in living PSB-D and BY-2 suspension cells was analyzed using confocal fluorescence microscopy. A clear FITC signal was observed in the lumen of the vacuoles of PSB-D cells as early as 5 min after exposure to OsRIP1-FITC (**Figure 2A**). Similar results were also observed for BY-2 cells although the fluorescence intensity was less at the early timepoints (**Figure 2B**). After treatment for 24 h, the fluorescence appeared to be more intense, and OsRIP1 conjugated with FITC exclusively partitioned in the vacuolar fraction, while the nuclei of PSB-D cells or BY-2 cells were clearly devoid of any fluorescence.

### 3.3 OsRIP1 caused a loss of plasma membrane integrity and vacuolar collapse in BY-2 cells but not in PSB-D cells

**Figure 3:**
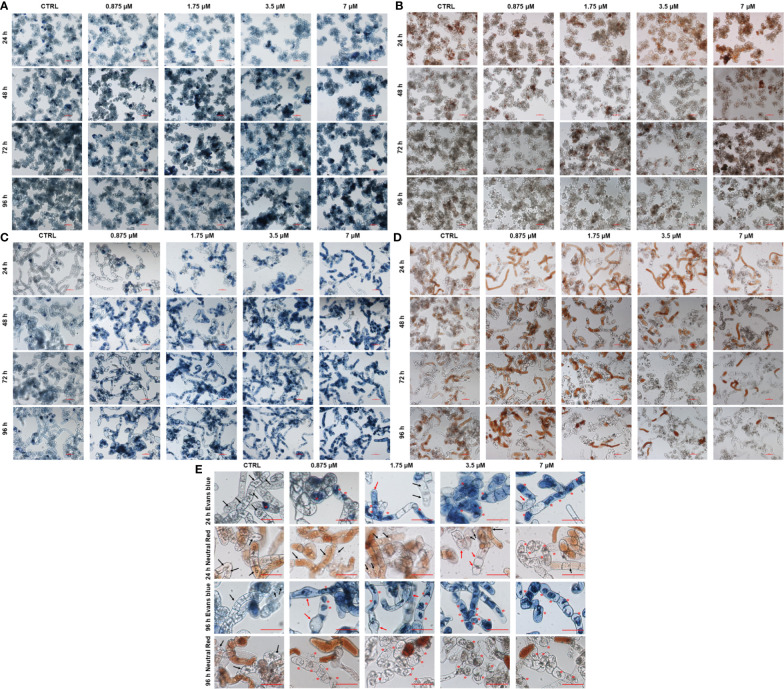
Light microscopy images of PSB-D cells **(A, B)** and BY-2 cells **(C, D)** stained with Evans blue **(A, C)** and neutral red **(B, D)** under a 10x objective. Cells were incubated in the absence or presence of 0.875-7 μM OsRIP1 for 24-96 h. All scale bars indicate 100 μm. **(E)** BY-2 cells stained with Evans blue and neutral red after 24 h or 96 h of different treatments, respectively. Asterisks (*) indicate vacuolar collapse and cell shrinkage; black arrows indicate normal nuclei; red arrows indicate cell swelling or plasmolysis.

After treatment with various concentrations of OsRIP1 (up to 7 μM) for 24-96 h, the integrity of the plasma membrane and the vital functions of vacuoles were estimated in PSB-D cells and BY-2 cells through the Evans blue and the neutral red staining, respectively. In OsRIP1-treated PSB-D cells, Evans blue uptake was similar as in the control group, with more intense cell staining when the incubation time was prolonged (**Figure 3A**). Most of the control cells were stained with neutral red, and a similar degree of neutral red uptake was also observed in PSB-D cells exposed to OsRIP1 (**Figure 3B**). No morphological changes were observed between control cells and OsRIP1-treated PSB-D cells. However, in OsRIP1-exposed BY-2 cells, the cell content was clearly disorganized, with plasmolysis and cell shrinkage. After 24 h, more than 50% of the BY-2 cells challenged with 3.5 μM OsRIP1 and nearly 90% of the cells challenged with 7 μM OsRIP1 were heavily stained, especially in the nucleus, whereas most control cells were not stained with Evans blue, with normal nuclei (**Figures 3C, E**). Evans blue uptake in BY-2 cells occurred in a time-dependent manner under OsRIP1 treatment. After 96 h treatment with 0.875 μM OsRIP1, BY-2 cells were swollen and revealed heavy staining of the nucleus (**Figure 3C**). With increasing OsRIP1 doses (up to 7 μM), nuclear condensation was characteristic of the end stages of cell death in BY-2 cells (**Figure 3C, E**). Neutral red was taken up in a dose- and time- dependent manner in BY-2 cells after exposure to OsRIP1. After 96 h, nearly 50% of the cells treated with 0.875 μM OsRIP1 were stained with neutral red, while only few cells with neutral red staining were detected after incubation with 1.75-7 μM OsRIP1. Most control cells were stained in red or pale red color. OsRIP1 treatment of BY-2 cells at 1.75-7 μM caused the decline in the cellular uptake of neutral red with longer incubation times, especially at 96 h when the majority of unstained cells with neutral red was shown to carry collapsed vacuoles and condensed nuclei as shown by staining with Evans blue (**Figures 3D, E**).

### 3.4 OsRIP1 decreased the fresh weight of BY-2 cells, independent of H_2_O_2_ production

**Figure 4:**
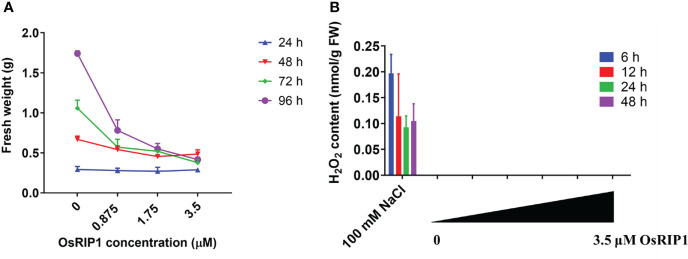
Responses of BY-2 cells to different concentrations of OsRIP1. **(A)** fresh weight of tobacco BY-2 cells harvested after treatment. **(B)** H_2_O_2_ levels in BY-2 cells exposed to different concentrations of OsRIP1. BY-2 cells treated with 100 mM NaCl were used as positive controls for the H_2_O_2_ determination assays. Four biological replicates and technical triplicates were performed for BY-2 cells treated in the absence (CTRL) or presence of OsRIP1 for desired incubation times. Data are expressed as mean ± standard deviation (SD).

The fresh weight of BY-2 pellets after OsRIP1 treatment was recorded (**Figure 4A**), and showed that with increasing concentrations of OsRIP1, fresh weight of samples was not changed at 24 h but it decreased slightly at 48 h compared to that of control cells. A significant reduction in fresh weight was shown at 72 h and 96 h for OsRIP1-treated BY-2 cells when compared with the control group. A clear increase in fresh weight of BY-2 cells was found after incubation with 0-0.875 μM OsRIP1 for 24-96 h. At the higher OsRIP1 concentrations (>1.75 μM) the fresh weight of the cells hardly changed over 48-96 h.

H_2_O_2_ accumulation is an early hallmark for a triggered immune response. For instance, the addition of 100 mM NaCl caused a significant increase in H_2_O_2_ production in BY-2 cells after 6-48 h. However, no accumulation of intracellular H_2_O_2_ was detected in BY-2 cells after exposure to 0-3.5 μM OsRIP1 (**Figure 4B**).

### 3.5 OsRIP1 caused RNA degradation and DNA fragmentation in BY-2 cells

**Figure 5:**
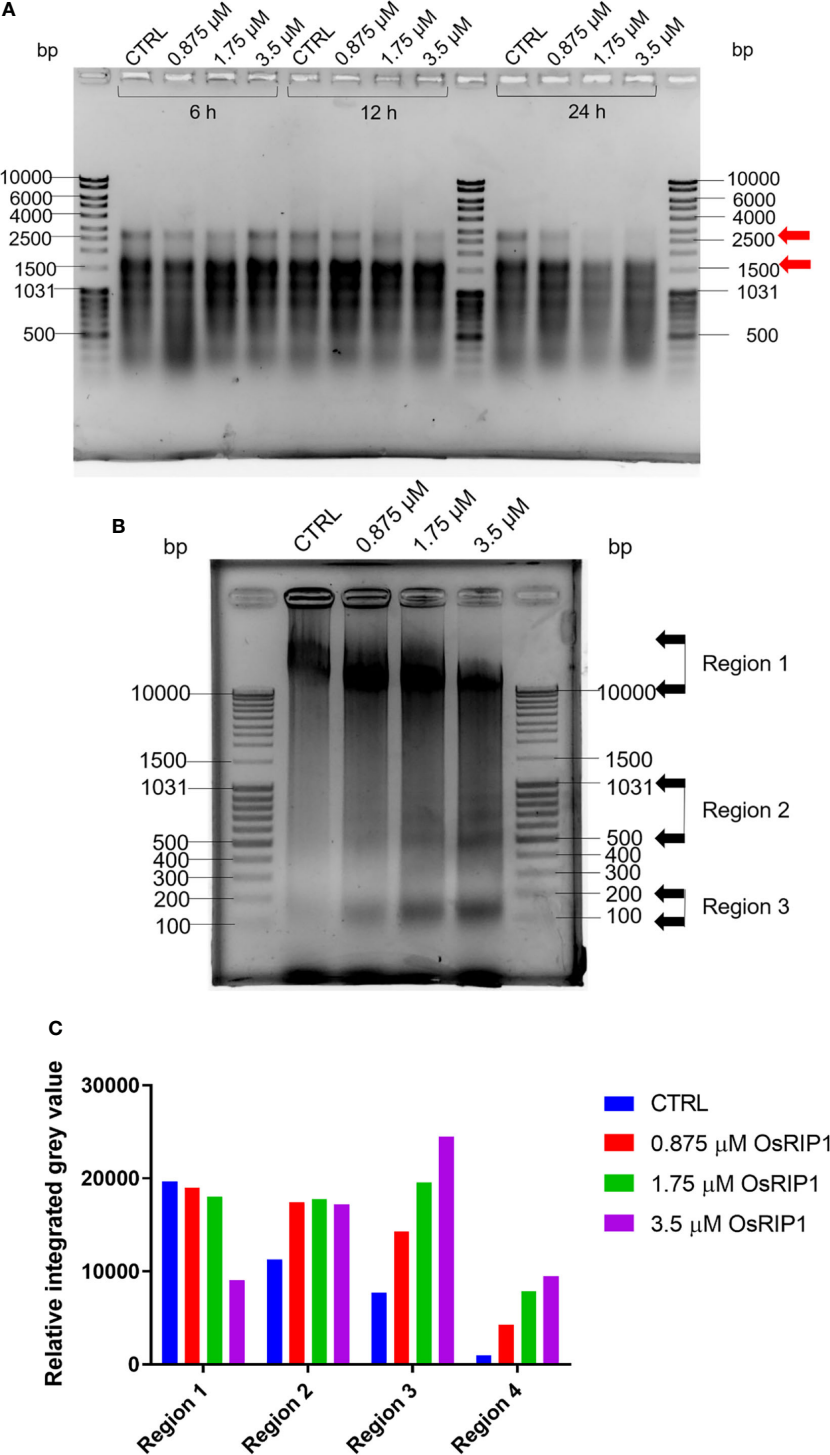
Agarose gel electrophoresis for nucleic acid samples of cultured tobacco BY-2 cells treated with OsRIP1 at 0 (CTRL), 0.875, 1.75, 3.5 μM. **(A)** 1 μg of total RNA. **(B)** Genomic DNA (11.693 μg) extracted from tobacco BY-2 cells (representative samples from three independent experiments) at 48 h after control (CTRL) or OsRIP1 treatment. MassRuler DNA Ladder Mix (5 μl, Thermo Scientific) were loaded in the gels. **(C)** Quantitative analysis of regions indicated with grey arrows in **(B)** using the software ImageJ in grayscale mode.

Total RNA was extracted from BY-2 cells exposed to OsRIP1 for 6 h, 12 h and 24 h, respectively, and visualized by electrophoresis (**Figure 5A**). The effect of OsRIP1 treatment on total RNA was clearer with longer exposure time. After OsRIP1 treatment for 24 h, at 1.75 μM and 3.5 μM RNA patterns showed more degradation, especially for fragments of approximately 2800 bp and those ranging from 1500-2000 bp (**Figure 5A**). Additionally, total DNA was isolated from control cells or cells treated with increasing concentrations of OsRIP1 for 48 h, respectively, and the integrity of DNA was evaluated by electrophoresis (**Figure 5B**). The majority of the genomic DNA extracted from the control cells migrated as an unresolved high molecular weight band of more than 10 kbp in length. On the contrary, a ladder pattern appeared in DNA from cells treated with increasing concentrations of OsRIP1 for 48 h. To quantify the differences between control and treated cells, four regions in the DNA pattern were selected and their band densities were calculated as relative integrated grey values (RIGV). Fragments in Region 1 above 10 kbp decreased with increasing doses of OsRIP1 (0.875-3.5 μM), while RIGV in Region 2 increased after OsRIP1 treatment compared to that of control cells, revealing that DNA degradation happened in BY-2 cells exposed to OsRIP1. Additionally, three bands in Region 3 (400-1031 bp) and the band (100-200 bp) in Region 4 were more visible in DNA extracted from cells treated with 3.5 μM OsRIP1, which was in line with increased RIGV in Region 3 and Region 4 with higher concentrations of OsRIP1 (**Figure 5C**).

### 3.6 Transcript levels of genes of interest quantified by RT-qPCR analysis

**Figure 6:**
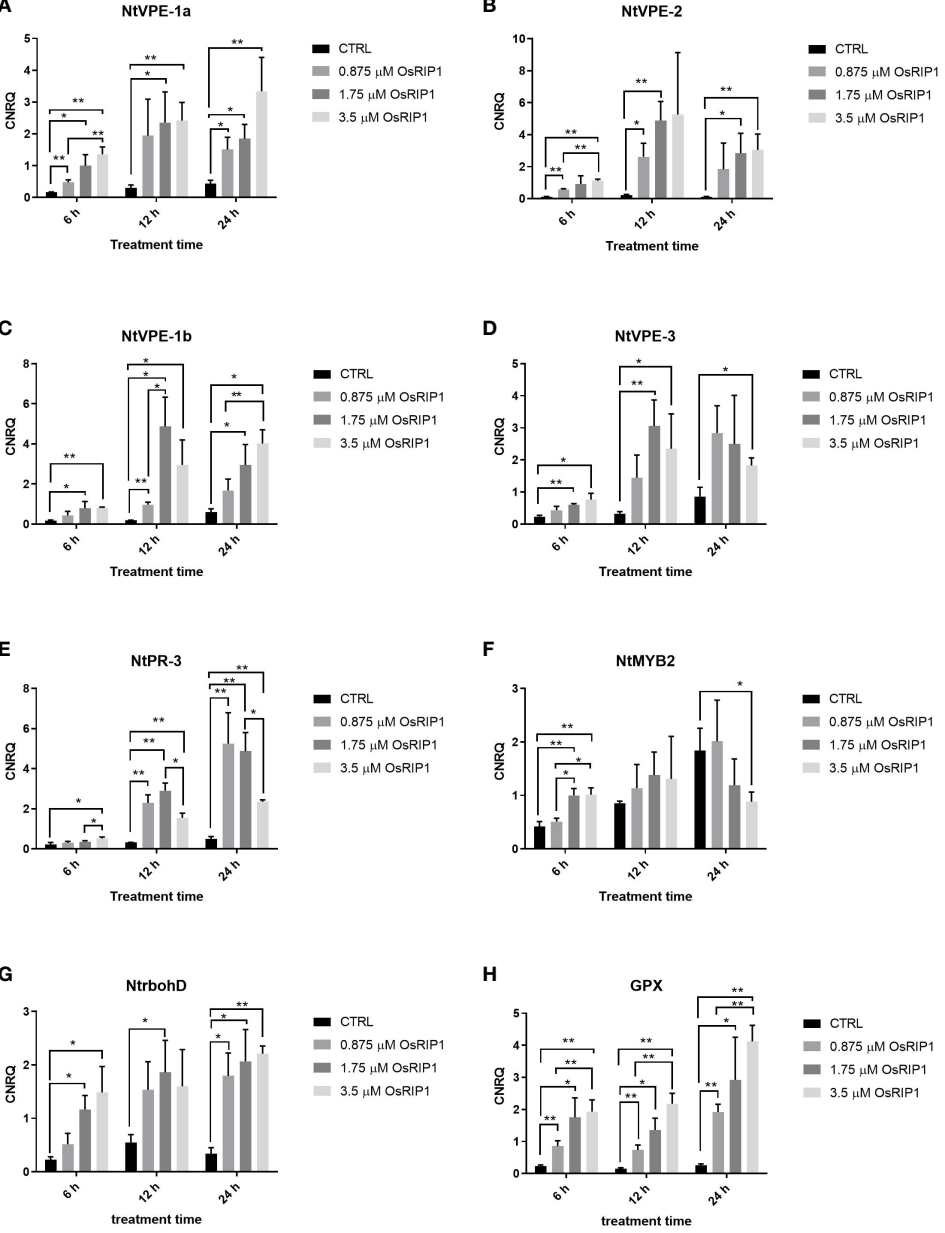
Relative expression levels (calibrated normalized relative quantities, CNRQ values) of genes of interest determined by RT-qPCR. Values are the mean ± SD of three independent experiments (n = 3). Asterisks indicate significant differences, which was determined by Multiple *t*-test, *p < 0.05; **p < 0.01. **(A)** NtVPE-1a. **(B)** NtVPE-2. **(C)** NtVPE-1b. **(D)** NtVPE-3. **(E)** NtPR-3. **(F)** NtMYB2. **(G)** NtrbohD. **(H)** GPX.

The transcript levels for four *VPE* genes of tobacco (*NtVPE-1a*, *NtVPE-1b*, *NtVPE-2*, *NtVPE-3*) were analyzed in BY-2 cells exposed to OsRIP1 for 6 h, 12 h and 24 h, respectively. The expression of these VPE genes was induced by the supplementation of OsRIP1 over time (**Figure 6**). The mRNA levels were weakly up-regulated at 6 h, but were strongly activated at 12 h. After 24 h, transcript levels for these 4 VPE genes declined as compared to 12 h after treatment for each concentration of OsRIP1. In addition, OsRIP1 treatment induced expression of all NtVPE genes in a dose-dependent manner.

Transcript levels for tobacco defense-related genes (*NtPR-3*, *NtMYB2*) and oxidative stress-related genes (the plasma membrane oxidase, *NtrbohD*; glutathione peroxidase, *GPX*) were also monitored (**Figure 6**). After 6 h of 3.5 μM OsRIP1 treatment, transcript levels for NtPR-3 were slightly up-regulated and NtMYB2 was stimulated more than 2-fold. Transcripts of NtrbohD and GPX were strongly up-regulated more than 6-fold and 8-fold in BY-2 cells exposed to 3.5 μM OsRIP1 for 6 h, respectively. Up-regulation of NtPR-3 mRNA levels triggered by 3.5 μM OsRIP1 was even less than that observed at 0.875 μM or at 1.75 μM OsRIP1 after 12 h or 24 h. Each concentration of OsRIP1 significantly induced NtPR-3, NtrbohD and GPX expression after 24 h, but there were no significant changes in the transcript level of NtMYB2 after 24 h incubation with OsRIP1. These data showed both concentration- and time- dependent stimulatory effects of OsRIP1 treatment on GPX expression during 24-96 h.

### 3.7 Necrotic lesions were formed after OsRIP1 infiltration in tobacco leaves

**Figure 7:**
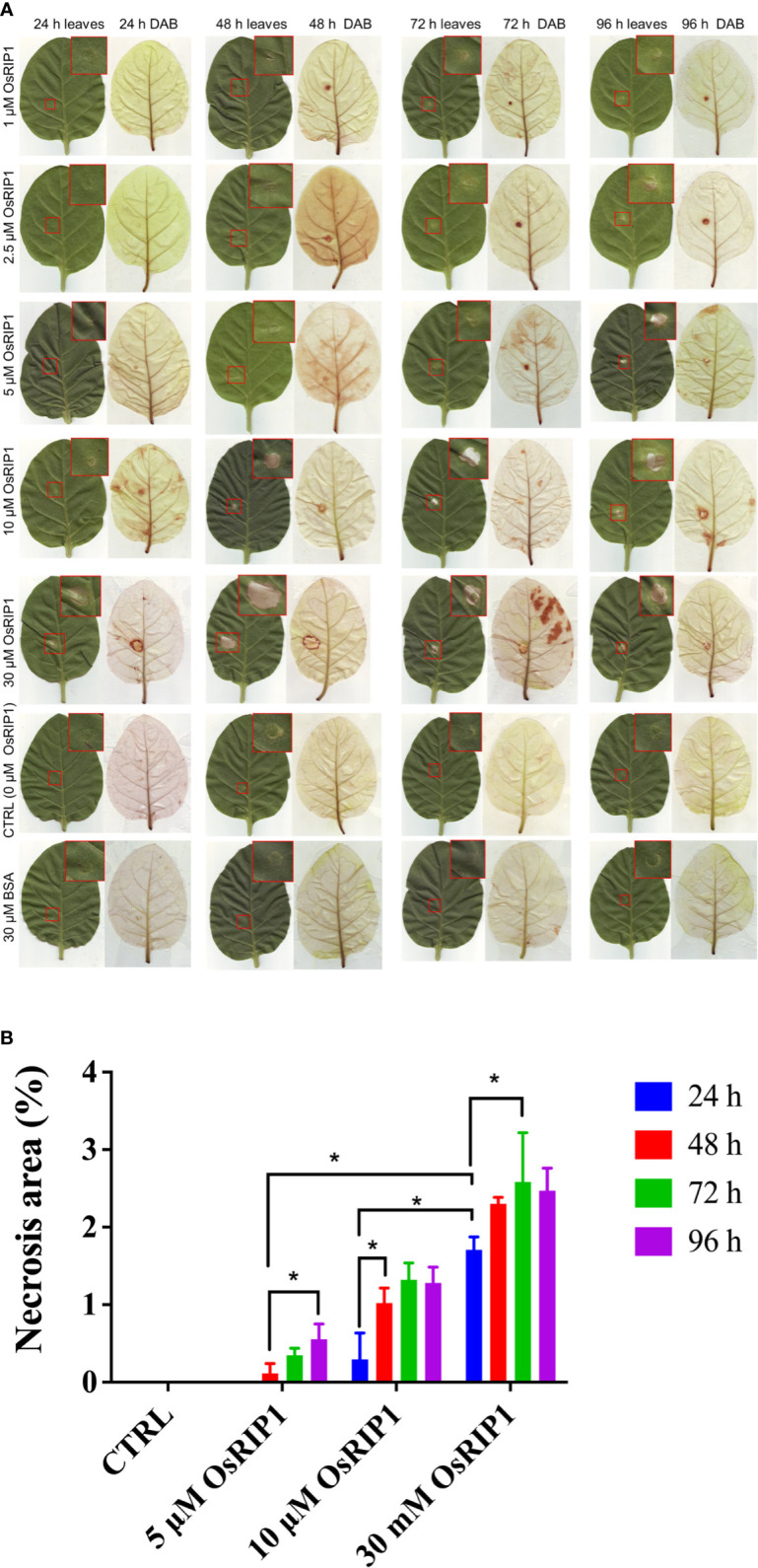
Leaves of 7-week-old Samsun NN tobacco plants were infiltrated with 50 mM potassium phosphate buffer (CTRL), with 30 μM BSA or with various concentrations of OsRIP1. **(A)** images of leaves after infiltration and chlorophyll removal after DAB staining. Brown DAB precipitates are indicative of the ROS burst. **(B)** necrotic area (% of the total leaf area) at different induction times post CTRL, BSA or OsRIP1 infiltration. At least three biological replicates were performed (n ≥ 3) for each treatment. Asterisks (*) indicate statistical difference (p < 0.05).

To study whether OsRIP1 is toxic to tobacco cells *in vivo*, different concentrations of OsRIP1 (1-30 μM) were infiltrated in leaves of 7-week-old *Nicotiana tabacum* cv. Samsun NN plants. The extent of necrosis at the site of injection expanded in a dose-dependent manner. The injury after ~10 μM OsRIP1 treatment was hardly visible at 24 h after infiltration. In contrast, the infiltration of 30 μM OsRIP1 for 24 h yielded a clear lesion (**Figure 7A**), representing 1.71% of the total leaf area, which is significantly different from other treatments at 24 h (**Figure 7B**). The brownish regions detected by DAB were present but confined to the size of injection area of tobacco leaves at 24-96 h after ~2.5 μM OsRIP1 infiltration (**Figure 7A**). The necrotic area resulting from the injection of 5 μM OsRIP1 showed a time-dependent increase, accompanied by limited H_2_O_2_ levels even at 96 h after infiltration. At the concentration of 10 μM, OsRIP1 caused a significant increase in the areas of necrosis at 48 h (1.02%) after infiltration compared to that at 24 h (0.29%) (**Figure 7B**). 30 μM OsRIP1 caused the lesion with the most severe necrosis (2.59%) at 72 h following infiltration and was significantly different from the effect observed at 24 h (**Figure 7B**). Infiltration of tobacco leaves with 30 μM OsRIP1 yielded limited H_2_O_2_ production confined to the border of the necrotic area, even up to 96 h. In contrast, no lesion area or H_2_O_2_ accumulation was detected in tobacco leaves after infiltration with 50 mM potassium phosphate buffer or with 30 μM BSA (**Figure 7A**).

## 4 Discussion

To study the effect of exogenously applied OsRIP1 on cell suspension cultures, cell viability and cell density at OD_600_ were determined. OsRIP1 caused cell death in tobacco BY-2 cells but not in *Arabidopsis* PSB-D cells, the former cell line being more susceptible to OsRIP1 exposure in a dose-dependent manner in terms of cell viability. Quantification of cell viability was consistent with the data of cell density at OD_600_, and strongly suggested that cell death in BY-2 cells was triggered by OsRIP1 after 24 h treatment and coincided with both the loss of integrity of the plasma membrane and vacuolar staining (**Figure 3**). The loss of neutral red uptake in many BY-2 cells was concomitant to a severe increase in Evans blue uptake, and these dead cells displayed a series of morphological changes, such as cell swelling at early stages, collapsed vacuoles, and nuclear condensation (**Figures 3C–E**). In contrast, PSB-D cells showed no morphological changes in response to OsRIP1 treatment (**Figures 3A, B**). These data are indicative of differential effects of OsRIP1 treatment on the growth of plant cells. To date, there are limited reports on the toxicity of RIPs to plant cell cultures. Grasso et al. (1980) originally described the toxicity of PAP for tobacco protoplasts, and this was confirmed by the subsequent discovery that high-level expression of PAP was harmful to transgenic tobacco (Lodge et al., 1993). Furthermore, PAP exerted inhibitory effects on the growth of carrot cells, but stimulated the growth of rice cells, while ricin and gelonin significantly stimulated the growth of both rice and carrot cells in liquid cultures (Battelli et al., 1984). These observations of the stimulating effect of RIPs on the growth of some plant cells lead to the hypothesis that RIPs may play a role in cell multiplication. Oppositely, in our study OsRIP1 exposure was demonstrated to be lethal to tobacco BY-2 cells, but not to *Arabidopsis* PSB-D cells. Therefore, different RIPs behave differentially towards various cell types. Furthermore, evaluating the effect of RIPs from rice on heterologous plant species may shed light on the question of whether these natural phytotoxins with biological activity could be considered promising candidates for the development of natural herbicides that use new modes of action in both natural and well-controlled environments.

The toxicity of certain RIPs for tobacco cells is somewhat correlated with the phenotype of transgenic tobacco plants overexpressing the RIPs. The ectopic expression of four type 2 RIPs from *Sambucus nigra*, SNA-I, SNA-I′, SNA-V and SNLRP, did not affect the growth and fertility of transgenic tobacco (Samsun NN) plants (Chen et al., 2002; Vandenbussche et al., 2004). However, overexpression of IRAb, a type 2 RIP from *Iris*, in tobacco, providing strong antiviral protection against tobacco mosaic virus (TMV) and TEV, resulted in impaired growth and development indicating that this type 2 RIP exerts a cytotoxic effect *in planta*. Similarly, not all type 1 RIPs are toxic to plants harboring the transgene. PAP (Lodge et al., 1993), PAP II (Wang et al., 1998) and type 3 RIP JIP60 (Görschen et al., 1997) are toxic to plant cells. Conversely, IRIP, a type 1 RIP from iris bulbs, did not provoke apparent phenotypic side effects, and thus was considered not to be cytotoxic to tobacco cells (Desmyter et al., 2003). All the above type 1 RIPs, either toxic or nontoxic to host cells, enhanced plant resistance against different viruses significantly. Likewise, transgenic tobacco plants overexpressing OsRIP1 showed less severe symptoms after infection with TMV compared to WT plants (De Zaeytijd, 2019). Furthermore, no symptoms of cell death were observed when OsRIP1 was expressed in *N. benthamiana* leaves by transient transformation of epidermal cells, and successful stable transformation of PSB-D cells with the OsRIP1-eGFP construct was also achieved (De Zaeytijd et al., 2019), indicating that the endogenously produced cytosolic OsRIP1 is not toxic to *N. benthamiana* epidermal cells and appears not to act on plant ribosomes. These findings suggesting the lack of cytotoxicity of OsRIP1 strikingly contrast with the case of OsRIP12, another type 1 RIP from rice. OsRIP12 is synthesized with a signal peptide and showed detrimental effects when ectopically expressed in either tobacco plants or PSB-D cells (De Zaeytijd, 2019).

In the present study, exogenous application of purified recombinant OsRIP1 to tobacco BY-2 cells showed OsRIP1-driven cell death in tobacco suspension cells, revealing a noteworthy difference between the *in vitro* cell system and the *in vivo* plant system. The presence of OsRIP1 in subcellular compartments may account for these discrepancies. Transport of OsRIP1 in plants may be different when the RIP is applied exogenously compared to when it is synthesized by the plant cells. To track the localization of OsRIP1, FITC labelling was employed. OsRIP1-FITC was internalized to the vacuolar lumen, but was not transported to nuclei in PSB-D cells and BY-2 cells (**Figure 2**). On the contrary, in the case of endogenous production of OsRIP1, subcellular localization studies revealed that OsRIP1 coupled to EGFP resided in the nucleus and the cytosol when expressed transiently in *N. benthamiana*, or *Arabidopsis* PSB-D suspension cells (De Zaeytijd et al., 2019). Differential effects between exogenous and endogenous OsRIP1 on plant cell fates may therefore be attributed to different transport pathways and subsequent differences in localization of OsRIP1.

Most type 1 RIPs are synthesized as precursor proteins containing a signal peptide, they were translocated to the endoplasmic reticulum and follow the secretory route, being targeted to the vacuole or the intracellular space, as described for saporin and PAP (Marshall et al., 2011). Conversely, OsRIP1 lacks a signal peptide and subcellular localization analysis confirmed its cytoplasmic localization (De Zaeytijd et al., 2019). It should be noted that those RIPs sorted to protein storage vacuoles are mostly catalytically inactive, as exemplified by ricin (Ashfaq et al., 2018). Exogenous application of OsRIP1 with enzymatic activity revealed the accumulation of OsRIP1 in vacuoles of tobacco BY-2 cells, and cells underwent vacuole-mediated programmed cell death (PCD). It is well documented that the vacuole functions as a key organelle in stress responses. Additionally, disintegration of the vacuolar membranes is a crucial event in plant cell death (Sampaio et al., 2022). Similar to cryptogein-induced PCD in tobacco BY-2 cells (Higaki et al., 2007), OsRIP1 treatments led to the collapse of the vacuolar membrane followed by the irreversible loss of plasma membrane integrity and cell shrinkage (**Figure 3**). These morphological observations imply that OsRIP1 triggered the vacuolar processing enzyme (VPE)-dependent cell death through destruction of vacuoles. These data are consistent with the observations of OsRIP1-induced transcript accumulation of VPE genes (*NtVPE-1a*, *NtVPE-1b*, *NtVPE-2*, *NtVPE-3*) (**Figure 6**). These caspase 1-like enzymes function as the executioner of a plant-specific PCD through disrupting vacuoles in response to abiotic stresses, and in pathogenesis as well as seed development (Mendes et al., 2013). This type of cell death results in the release of vacuolar hydrolytic enzymes into the cytosol, and is considered as an efficient defense against viruses proliferating in the cytosol, as described for the tobacco mosaic virus (Hatsugai et al., 2004). The destruction of vacuoles also leads to the degradation of various organelles (i.e., the nucleus) *via* the action of releasing enzymes (i.e., nucleases and proteinases), consequently provoking cell death. Moreover, OsRIP1 treatment of tobacco BY-2 cells induced an increase in transcripts of tobacco defense-related genes NtPR-3 and NtMYB2 as compared to control cells at 6 h after treatments (**Figure 6**), suggestive of the involvement of cell defense mechanisms.

DNA fragmentation is a further invariable event of cell death resulting from pathogen infection or other stresses in animals and plants. Oligonucleosomal units were observed after treatments with different concentrations of OsRIP1 (0.875, 175, 3.5 μM) and the formation of a DNA ladder was clearly visible with increasing concentrations of OsRIP1 at 48 h after treatments (**Figure 5B**). OsRIP1 induction of RNA degradation in BY-2 cells was observed at 24 h after treatment, whereas 6 h of OsRIP1 exposure significantly altered the transcript levels for genes of interest (**Figures 5A**, **6**). A higher degree of DNA fragmentation occurred in BY-2 cells treated with elevated concentrations of OsRIP1. Small fragments of multiples of 180-200 bp in size were observed (**Figure 5C**), which is characteristic of internucleosomal cleavage of nuclear DNA into nucleosomal fragments resulting from the activation of an endonuclease(s) (Oberhammer et al., 1993). Conversely, a previous study in animal cells showed that the application of 4 μM OsRIP1 to HeLa cells did not induce DNA fragmentation (Chen et al., 2021). Furthermore, OsRIP1 preferably accumulated at the plasma membrane of HeLa cells, whereas OsRIP1 was internalized into intracellular vacuoles of plant cells (**Figure 2**). These observations suggest that the mode of action underlying OsRIP1-driven cell death may differ between human and plant cells. These findings also clearly point towards a common characteristic for OsRIP1 induced cell death in BY-2 cells and HeLa cells, OsRIP1 being separated from ribosomes. Indeed, the activity of recombinant OsRIP1 on translation inhibition was experimentally confirmed using the *in vitro* translation system from rabbit reticulocytes (De Zaeytijd et al., 2019). Taken into account that recombinant OsRIP1 possesses enzymatic activity, these data imply that OsRIP1 is capable of killing BY-2 cells independent of its catalytic activity on host ribosomes. The phenomenon that RIP-induced cell death is independent of translation inhibition was also described for other RIPs, such as α-sarcin (Olmo et al., 2001), saporin (Sikriwal et al., 2008), stenodactylin (Polito et al., 2016) and kirkiin (Bortolotti et al., 2021).

Reactive oxygen species (ROS) are typical signaling molecules in living cells that participate in a wide variety of biochemical and physiological processes (Mignolet-Spruyt et al., 2016). In this study, no H_2_O_2_ accumulation was detected in tobacco BY-2 cells exposed to OsRIP1 for 6-48 h (**Figure 4**). Consistent with that, application of exogenous OsRIP1 on the tissue level indicated the limited amounts of H_2_O_2_ production associated with lesion formation (**Figure 7**). These results clearly suggest that OsRIP1-induced cell death is independent of H_2_O_2_ production. The plasma membrane NADPH oxidase NtrbohD is responsible for ROS production in elicited tobacco cells (Simon-Plas et al., 2002), as described for the inducers of antibiotic filipin (Bonneau et al., 2010) and cryptogein (Coursol et al., 2015). NtrbohD was transcriptionally up-regulated by OsRIP1 treatment. OsRIP1-induced stimulation of GPX expression was much higher than that of NtrbohD (**Figure 6**), indicative of the suppression of ROS production. It implies that activation of antioxidant enzymes may depress the ROS burst in OsRIP1-treated BY-2 cells and it may account for the absence of cellular ROS accumulation (**Figure 4**). Although H_2_O_2_ was a characteristic signal involved in HR and systemic acquired resistance to induce PCD, it may not be indispensable for the execution of cell death program. External application of C2-ceramide caused H_2_O_2_ production and induction of PCD, but this sphingolipid-induced PCD was independent of the generation of ROS in *Arabidopsis* cell suspension cultures (Townley et al., 2005) or in tobacco BY-2 cells (Lachaud et al., 2011). Yun et al. (2011) also reported that the cell death in response to incompatible pathogen infections in the gsnor1-3 mutant is due to high S-nitrosothiol concentrations and is salicylic acid- and H_2_O_2_-independent. Thus, cell death is not necessarily accompanied by H_2_O_2_ formation.

## 5 Conclusion

This study indicates that OsRIP1, a type 1 cytosolic RIP, triggered cell death in tobacco BY-2 cells but not in *Arabidopsis* PSB-D cells. OsRIP1 treatment of BY-2 cells caused a loss of integrity of the plasma membrane, vacuolar collapse, RNA degradation and DNA fragmentation, without the accumulation of H_2_O_2_. Moreover, visible necrosis and a limited HR were induced by infiltration of high concentrations of OsRIP1 in epidermal cells of Samsun NN tobacco plants. Vacuolar collapse, the up-regulation of VPE genes and the evidence for OsRIP1 targeting to plant vacuoles clearly suggest that OsRIP1 triggers VPE-dependent cell death through the destruction of vacuoles in tobacco BY-2 cells, independent of its *N*-glycosylase activity on cytosolic ribosomes. The precise mechanism of OsRIP1-induced cell death in tobacco BY-2 cells remains to be elucidated.

## Data availability statement

The original contributions presented in the study are included in the article/**Supplementary Material**. Further inquiries can be directed to the corresponding author.

## Author contributions

SC and ED conceived and designed the experiments. SC, KG, and IV performed the experiments. ED supervised the experiments. SC wrote the manuscript, KG, IV revised the manuscript, and ED edited and finalized the manuscript. All authors contributed to the article and approved the submitted version.

## Funding

This work was supported in part by Guangzhou Elite Project and Fund for Scientific Research - Flanders.

## Acknowledgments

This work was supported in part by Guangzhou Elite Project and Fund for Scientific Research - Flanders.

## Conflict of interest

The authors declare that the research was conducted in the absence of any commercial or financial relationships that could be construed as a potential conflict of interest.

## Publisher’s note

All claims expressed in this article are solely those of the authors and do not necessarily represent those of their affiliated organizations, or those of the publisher, the editors and the reviewers. Any product that may be evaluated in this article, or claim that may be made by its manufacturer, is not guaranteed or endorsed by the publisher.

## References

[B1] Ashfaq A. Soma Sekhar Reddy P. Kumar A. Selvaraj V. M. Dinesh Kumar V. (2018). “Ricin and RCA - the enemies within castor (Ricinus communis l.): A perspective on their biogenesis, mechanism of action, detection methods and detoxification strategies,” in The castor bean genome (Cham, Switzerland: Springer).

[B2] Barbieri L. Valbonesi P. Bonora E. Gorini P. Bolognesi A. Stirpe F. (1997). Polynucleotide: adenosine glycosidase activity of ribosome-inactivating proteins: effect on DNA, RNA and poly (A). Nucleic Acids Res. 25, 518–522. doi: 10.1093/nar/25.3.518 9016590 PMC146458

[B3] Battelli M. G. Lorenzoni E. Stirpe F. Cella R. Parisi B. (1984). Differential effect of ribosome-inactivating proteins on plant ribosome activity and plant cells growth. J. Exp. Bot. 35, 882–889. doi: 10.1093/jxb/35.6.882

[B4] Bolognesi A. Bortolotti M. Maiello S. Battelli M. G. Polito L. (2016). Ribosome-inactivating proteins from plants: A historical overview. Molecules 21, 1627. doi: 10.3390/molecules21121627 PMC6273060 27898041

[B5] Bonneau L. Gerbeau-Pissot P. Thomas D. Der C. Lherminier J. Bourque S.. (2010). Plasma membrane sterol complexation, generated by filipin, triggers signaling responses in tobacco cells. Biochim. Et. Biophys. Acta (BBA)-Biomembranes. 1798, 2150–2159. doi: 10.1016/j.bbamem.2010.07.026 20674542

[B6] Bortolotti M. Maiello S. Ferreras J. M. Iglesias R. Polito L. Bolognesi A. (2021). Kirkiin: A new toxic type 2 ribosome-inactivating protein from the caudex of *Adenia kirkii* . Toxins 13, 81. doi: 10.3390/toxins13020081 33499082 PMC7912562

[B7] Chen S. Lóssio C. F. Verbeke I. Verduijn J. Parakhonskiy B. van der Meeren L.. (2021). The type-1 ribosome-inactivating protein OsRIP1 triggers caspase-independent apoptotic-like death in HeLa cells. Food Chem. Toxicol. 157, 112590. doi: 10.1016/j.fct.2021.112590 34601042

[B8] Chen Y. Peumans W. J. Van Damme E. J. (2002). The sambucus nigra type-2 ribosome-inactivating protein SNA-i′ exhibits in planta antiviral activity in transgenic tobacco. FEBS Lett. 516, 27–30. doi: 10.1016/S0014-5793(02)02455-9 11959096

[B9] Chen M. Zeng H. Qiu D. Guo L. Yang X. Shi H.. (2012). Purification and characterization of a novel hypersensitive response-inducing elicitor from *Magnaporthe oryzae* that triggers defense response in rice. PloS One 7, e37654. doi: 10.1371/journal.pone.0037654 22624059 PMC3356297

[B10] Citores L. Iglesias R. Ferreras J. M. (2021). Antiviral activity of ribosome-inactivating proteins. Toxins 13, 80. doi: 10.3390/toxins13020080 33499086 PMC7912582

[B11] Coursol S. Fromentin J. Noirot E. Brière C. Robert F. Morel J.. (2015). Long-chain bases and their phosphorylated derivatives differentially regulate cryptogein-induced production of reactive oxygen species in tobacco (*Nicotiana tabacum*) BY-2 cells. New Phytol. 205, 1239–1249. doi: 10.1111/nph.13094 25303640

[B12] Desmyter S. Vandenbussche F. Hao Q. Proost P. Peumans W. J. Van Damme E. J. (2003). Type-1 ribosome-inactivating protein from iris bulbs: a useful agronomic tool to engineer virus resistance? Plant Mol. Biol. 51, 567–576. doi: 10.1023/a:1022389205295 12650622

[B13] Develtere W. (2019). Development of a screening assay for homologous recombination and optimization of the CRISPR/Cas9 system through promoter analysis (Ghent, Belgium: Ghent University).

[B14] De Zaeytijd J. (2019). Biological activity of ribosome-inactivating proteins from rice and their involvement in stress responses (Ghent, Belgium: Ghent University).

[B15] De Zaeytijd J. Rougé P. Smagghe G. Van Damme E. J. (2019). Structure and activity of a cytosolic ribosome-inactivating protein from rice. Toxins 11, 325. doi: 10.3390/toxins11060325 PMC6628440 31174339

[B16] Görschen E. Dunaeva M. Hause B. Reeh I. Wasternack C. Parthier B. (1997). Expression of the ribosome-inactivating protein JIP60 from barley in transgenic tobacco leads to an abnormal phenotype and alterations on the level of translation. Planta 202, 470–478. doi: 10.1007/s004250050151 9265788

[B17] Grasso S. Jones P. White R. (1980). Inhibition of tobacco mosaic virus multiplication in tobacco protoplasts by the pokeweed inhibitor. J. Phytopathol. 98, 53–58. doi: 10.1111/j.1439-0434.1980.tb03713.x

[B18] Hatsugai N. Kuroyanagi M. Yamada K. Meshi T. Tsuda S. Kondo M.. (2004). A plant vacuolar protease, VPE, mediates virus-induced hypersensitive cell death. Science 305, 855–858. doi: 10.1126/science.1099859 15297671

[B19] Higaki T. Goh T. Hayashi T. Kutsuna N. Kadota Y. Hasezawa S.. (2007). Elicitor-induced cytoskeletal rearrangement relates to vacuolar dynamics and execution of cell death: *in vivo* imaging of hypersensitive cell death in tobacco BY-2 cells. Plant Cell Physiol. 48, 1414–1425. doi: 10.1093/pcp/pcm109 17704529

[B20] Iglesias R. Citores L. Ragucci S. Russo R. Di Maro A. Ferreras J. M. (2016). Biological and antipathogenic activities of ribosome-inactivating proteins from *Phytolacca dioica* l. Biochim. Biophys. Acta (BBA)-General. Subj. 1860, 1256–1264. doi: 10.1016/j.bbagen.2016.03.011 26971856

[B21] Jia Q. Sun S. Kong D. Song J. Wu L. Yan Z.. (2020). Ectopic expression of Gs5PTase8, a soybean inositol polyphosphate 5-phosphatase, enhances salt tolerance in plants. Int. J. Mol. Sci. 21, 1023. doi: 10.3390/ijms21031023 PMC7037738 32033113

[B22] Kariya K. Demiral T. Sasaki T. Tsuchiya Y. Turkan I. Sano T.. (2013). A novel mechanism of aluminium-induced cell death involving vacuolar processing enzyme and vacuolar collapse in tobacco cell line BY-2. J. Inorgan. Biochem. 128, 196–201. doi: 10.1016/j.jinorgbio.2013.07.001 23891542

[B23] Keerio A. U. Nazir T. Anwar T. Zeeshan Majeed M. Abdulle Y. A. Jatoi G. H.. (2020). Sub-Lethal effects of partially purified protein extracted from *Beauveria bassiana* (Balsamo) and its presumptive role in tomato (*Lycopersicon esculentum* l.) defense against whitefly (*Bemisia tabaci* genn.). Insects 11, 574. doi: 10.3390/insects11090574 PMC7564989 32867017

[B24] Lachaud C. Da Silva D. Amelot N. Béziat C. Brière C. Cotelle V.. (2011). Dihydrosphingosine-induced programmed cell death in tobacco BY-2 cells is independent of H2O2 production. Mol. Plant 4, 310–318. doi: 10.1093/mp/ssq077 21199880

[B25] Leah R. Tommerup H. Svendsen I. Mundy J. (1991). Biochemical and molecular characterization of three barley seed proteins with antifungal properties. J. Biol. Chem. 266, 1564–1573. doi: 10.1016/S0021-9258(18)52331-0 1899089

[B26] Lodge J. K. Kaniewski W. K. Tumer N. E. (1993). Broad-spectrum virus resistance in transgenic plants expressing pokeweed antiviral protein. Proc. Natl. Acad. Sci. 90, 7089–7093. doi: 10.1073/pnas.90.15.7089 8346221 PMC47081

[B27] Mahjouri S. Kosari-Nasab M. Kazemi E. M. Divband B. Movafeghi A. (2020). Effect of Ag-doping on cytotoxicity of SnO2 nanoparticles in tobacco cell cultures. J. Hazard. Materials. 381, 121012. doi: 10.1016/j.jhazmat.2019.121012 31437804

[B28] Marshall R. S. D’avila F. Di Cola A. Traini R. Spanò L. Fabbrini M. S.. (2011). Signal peptide-regulated toxicity of a plant ribosome-inactivating protein during cell stress. Plant J. 65, 218–229. doi: 10.1111/j.1365-313X.2010.04413.x 21223387

[B29] Mendes G. C. Reis P. A. Calil I. P. Carvalho H. H. Aragão F. J. Fontes E. P. (2013). GmNAC30 and GmNAC81 integrate the endoplasmic reticulum stress-and osmotic stress-induced cell death responses through a vacuolar processing enzyme. Proc. Natl. Acad. Sci. 110, 19627–19632. doi: 10.1073/pnas.1311729110 24145438 PMC3845183

[B30] Mignolet-Spruyt L. Xu E. Idänheimo N. Hoeberichts F. A. Mühlenbock P. Brosché M.. (2016). Spreading the news: subcellular and organellar reactive oxygen species production and signalling. J. Exp. Bot. 67, 3831–3844. doi: 10.1093/jxb/erw080 26976816

[B31] Nagata T. Nemoto Y. Hasezawa S. (1992). Tobacco BY-2 cell line as the “HeLa” cell in the cell biology of higher plants. Int. Rev. Cytol. 132, 1–30. doi: 10.1016/S0074-7696(08)62452-3

[B32] Oberhammer F. Wilson J. W. Dive C. Morris I. Hickman J. A. Wakeling A. E.. (1993). Apoptotic death in epithelial cells: cleavage of DNA to 300 and/or 50 kb fragments prior to or in the absence of internucleosomal fragmentation. EMBO J. 12, 3679–3684. doi: 10.1002/j.1460-2075.1993.tb06042.x 8253089 PMC413644

[B33] Olmo N. Turnay J. De Buitrago G. G. De Silanes I. L. Gavilanes J. G. Lizarbe M. A. (2001). Cytotoxic mechanism of the ribotoxin α-sarcin: induction of cell death *via* apoptosis. Eur. J. Biochem. 268, 2113–2123. doi: 10.1046/j.1432-1327.2001.02086.x 11277935

[B34] Poborilova Z. Opatrilova R. Babula P. (2013). Toxicity of aluminium oxide nanoparticles demonstrated using a BY-2 plant cell suspension culture model. Environ. Exp. Bot. 91, 1–11. doi: 10.1016/j.envexpbot.2013.03.002

[B35] Polito L. Bortolotti M. Pedrazzi M. Mercatelli D. Battelli M. G. Bolognesi A. (2016). Apoptosis and necroptosis induced by stenodactylin in neuroblastoma cells can be completely prevented through caspase inhibition plus catalase or necrostatin-1. Phytomedicine 23, 32–41. doi: 10.1016/j.phymed.2015.11.006 26902405

[B36] Rustgi S. Pollmann S. Buhr F. Springer A. Reinbothe C. Von Wettstein D.. (2014). JIP60-mediated, jasmonate-and senescence-induced molecular switch in translation toward stress and defense protein synthesis. Proc. Natl. Acad. Sci. 111, 14181–14186. doi: 10.1073/pnas.1415690111 25225401 PMC4191784

[B37] Saghai-Maroof M. A. Soliman K. M. Jorgensen R. A. Allard R. (1984). Ribosomal DNA spacer-length polymorphisms in barley: Mendelian inheritance, chromosomal location, and population dynamics. Proc. Natl. Acad. Sci. 81, 8014–8018. doi: 10.1073/pnas.81.24.8014 6096873 PMC392284

[B38] Sampaio M. Neves J. Cardoso T. Pissarra J. Pereira S. Pereira C. (2022). Coping with abiotic stress in plants–an endomembrane trafficking perspective. Plants 11, 338. doi: 10.3390/plants11030338 35161321 PMC8838314

[B39] Sawasaki T. Nishihara M. Endo Y. (2008). RIP and RALyase cleave the sarcin/ricin domain, a critical domain for ribosome function, during senescence of wheat coleoptiles. Biochem. Biophys. Res. Commun. 370, 561–565. doi: 10.1016/j.bbrc.2008.03.124 18395011

[B40] Sikriwal D. Ghosh P. Batra J. K. (2008). Ribosome inactivating protein saporin induces apoptosis through mitochondrial cascade, independent of translation inhibition. Int. J. Biochem. Cell Biol. 40, 2880–2888. doi: 10.1016/j.biocel.2008.06.004 18611444

[B41] Simon-Plas F. Elmayan T. Blein J. P. (2002). The plasma membrane oxidase NtrbohD is responsible for AOS production in elicited tobacco cells. Plant J. 31, 137–147. doi: 10.1046/j.1365-313X.2002.01342.x 12121444

[B42] Surguchov A. Bernal L. Surguchev A. A. (2021). Phytochemicals as regulators of genes involved in synucleinopathies. Biomolecules 11, 624. doi: 10.3390/biom11050624 33922207 PMC8145209

[B43] Townley H. E. Mcdonald K. Jenkins G. I. Knight M. R. Leaver C. J. (2005). Ceramides induce programmed cell death in arabidopsis cells in a calcium-dependent manner. Biol. Chem. 386, 2. doi: 10.1515/BC.2005.020 15843160

[B44] Vandenbussche F. Desmyter S. Ciani M. Proost P. Peumans W. J. Van Damme E. J. (2004). Analysis of the in planta antiviral activity of elderberry ribosome-inactivating proteins. Eur. J. Biochem. 271, 1508–1515. doi: 10.1111/j.1432-1033.2004.04059.x 15066176

[B45] Velikova V. Yordanov I. Edreva A. (2000). Oxidative stress and some antioxidant systems in acid rain-treated bean plants: protective role of exogenous polyamines. Plant Sci. 151, 59–66. doi: 10.1016/S0168-9452(99)00197-1

[B46] Wang P. Zoubenko O. Tumer N. E. (1998). Reduced toxicity and broad spectrum resistance to viral and fungal infection in transgenic plants expressin pokeweed antiviral protein II. Plant Mol. Biol. 38, 957–964. doi: 10.1023/A:1006084925016 9869402

[B47] Wong J. H. Bao H. Ng T. B. Chan H. H. L. Ng C. C. W. Man G. C. W.. (2020). New ribosome-inactivating proteins and other proteins with protein synthesis–inhibiting activities. Appl. Microbiol. Biotechnol. 104, 4211–4226. doi: 10.1007/s00253-020-10457-7 32193575

[B48] Wytynck P. (2020). RIPs, a cereal killer?: the physiological role of rice ribosome-inactivating proteins (RIPs) in abiotic stress (Ghent, Belgium: Ghent University).

[B49] Wytynck P. Lambin J. Chen S. Demirel Asci S. Verbeke I. De Zaeytijd J.. (2021). Effect of RIP overexpression on abiotic stress tolerance and development of rice. Int. J. Mol. Sci. 22, 1434. doi: 10.3390/ijms22031434 33535383 PMC7867109

[B50] Wytynck P. Rougé P. Van Damme E. J. (2017). Genome-wide screening of oryza sativa ssp. japonica and indica reveals a complex family of proteins with ribosome-inactivating protein domains. Phytochemistry 143, 87–97. doi: 10.1016/j.phytochem.2017.07.009 28797946

[B51] Yun B.-W. Feechan A. Yin M. Saidi N. B. Le Bihan T. Yu M.. (2011). S-nitrosylation of NADPH oxidase regulates cell death in plant immunity. Nature 478, 264–268. doi: 10.1038/nature10427 21964330

