# Supplementary material for: Differential effects of the recombinant type 1 ribosome-inactivating protein, OsRIP1, on growth of PSB-D and BY-2 cells
Source: Front Plant Sci. 2022 Sep 29;13:1019591. doi: 10.3389/fpls.2022.1019591 (PMC9557087; doi:10.3389/fpls.2022.1019591)
Supplement: Supplementary file 1 [file Table_1.pdf]

Supplementary Material

Differential effects of the recombinant type 1 ribosome-inactivating protein, OsRIP1, on growth of PSB-D and BY-2 cells

Simin Chen, Koen Gistelinck, Isabel Verbeke, Els J.M. Van Damme

1. Supplementary Tables

Table S1. Primers used for RT-qPCR analysis and their efficiencies

Category	Gene	Primer sequence		Size of amplicon	Efficiency	Correlation coefficients (R^2)
Vacuolar processing enzyme (VPE genes) genes	NtVPE-1a	FP	GCCGCCCCTACCTTTGATA	92 bp	1.971 +/- 0.083	0.970
		RP	ACAGAGTACCGGCAATGTGAC			
	NtVPE-1b	FP	AGAGAACCCAAGACGAGGAGT	156 bp	1.953+/- 0.063	0.980

		RP	TTTCCGCTGCCTCCACTAAG			
	NtVPE-2	FP	AAATGGGCTGTGTTGGTTGC	71 bp	1.802 +/- 0.037	0.988
		RP	GCGTGACAAACATCTGCCTG			
	NtVPE-3	FP	AGTTCCCTCCACAGGGGTTT	198 bp	1.817 +/- 0.030	0.992
		RP	ACAGAGCAACAATTACAACCAGG			
Tobacco defense-related genes	NtPR-3	FP	CATCATCAATGGGGGATTGG	139 bp	1.933 +/- 0.033	0.991
		RP	AAAAGACCTCTGGTTGCCGC			
	NtMYB2	FP	GGTATGGAGGGTACCGGTGG	228 bp	1.949 +/- 0.113	0.936
		RP	GTTGACTCCCAATAATTAGCTGGC			
Genes related to enzymes associated with oxidative stress	Plasma membrane NADPH oxidase, NtrbohD	FP	CATCAAAACAGCTAAGGACACAG	61 bp	1.802 +/- 0.017	0.997
		RP	TACACAATAGGGAGAGTTGGTAG			
	Glutathione peroxidase (GPX)	FP	TGCAACTCGTGTTCTCATACCA	111 bp	1.907 +/- 0.034	0.991
		RP	ACTGTTGGAATCGGAGGAGT			

Reference genes	Actin-7	FP	ATGCCTATGTGGGTGACGAAG	197 bp	1.986 +/- 0.015	0.997
		RP	TCTGTTGGCCTTAGGGTTGAG			
	EF1α	FP	GTGGAAGTTTGAGACCACCAA	216 bp	2.086 +/- 0.077	0.971
		RP	ATCATTTGCTTGACACCAAGG			

FP: Forward Primer; RP: Reverse Primer
